# Longitudinal Associations of Electroconvulsive Therapy with All-cause Mortality and Suicide Deaths in Depression and Other Psychiatric Disorders: A Systematic Review and Meta-Analysis

**DOI:** 10.21203/rs.3.rs-6180102/v1

**Published:** 2025-03-31

**Authors:** Taeho Greg Rhee, sungryul shim, Madeeha Nasir, Roger McIntyre, Tyler Kaster, Samuel Wilkinson

## Abstract

**Objective:** Electroconvulsive therapy (ECT) is among the most effective treatments for mood disorders and other psychotic disorders. This study meta-analyzed the effects of ECT on all-cause mortality and suicide deaths using longitudinal studies. **Methods:** PubMed/MEDLINE, PsycINFO, Cochrane Library, Embase, and Google Scholar were searched from inception through January 21, 2025, with no language limits. Inclusion criteria were as follow: (1) patients with diagnoses of mental disorders; (2) intervention consisted of ECT compared with placebo, usual care or another intervention; (3) all-cause mortality and suicide deaths as outcome measures; and (4) clinical trial or longitudinal cohort study designs where the aforementioned interventions preceded the observations of outcome measures. Adjusted hazard ratio [HR] with their corresponding 95% confidence intervals (CIs) were calculated using fixed- or random-effects models. Moderator analyses were also performed. **Results:** Overall, 17 studies consisting of 1,182,501 individuals (n=40,867 for ECT, n=1,141,634 for those receiving comparable interventions) were included. ECT was associated with a reduction in risk of all-cause mortality (HR, 0.70 [95% CI, 0.61-0.81]; *p* <0.001), a finding that was consistent at 3 months, 6 months, and 12 months of follow-up. Sex demonstrated a very small moderating effect on this relationship, with ECT being slightly less protective against mortality risk for females compared to males (standardized beta coefficient, -0.01 [-0.01 to 0.00]; *p* =0.036). Regions also had a moderating effect ( *p* =0.002). Japan had the largest effect size (HR, 0.17 [0.04-0.72]) and Denmark had the smallest (HR, 0.87 [0.83-0.92]). ECT was associated with a reduction in suicide risk at 3 months of follow-up (HR, 0.53 [0.39-0.72]; *p* <0.001) but not at 1, 6, or 12 months of follow-up. **Conclusions:** ECT is associated with a reduced risk of all-cause mortality. ECT, however, was not consistently associated with a reduced risk of suicide.

### Introduction

Mood disorders are a leading cause of disability and mortality.[1] Mood disorders are associated with a dramatically reduced life expectancy, translating to approximately 10 years of lost life expectancy among males and 7–11 years among females.[2] Those who suffer from mood disorders are at an increased risk of suicide when compared to the general population. However, increased rates of mortality among this group are not solely attributable to suicide; risks for deaths due to cardiovascular disease, cancer, and other comorbid medical conditions are also elevated among individuals with mood disorders.[3–5] Individuals with schizophrenia spectrum and other psychotic disorders are also at considerably elevated risk of premature mortality (including suicide) compared to the general population.[6]

Electroconvulsive therapy (ECT) is among the most effective treatments for mood disorders,[7] and also has efficacy for treatment-resistant psychotic disorders.[8] Prior research suggests that ECT may reduce the risk of suicide as well as all-cause mortality. However, several methodological limitations cast uncertainty over this conclusion. For instance, one early study followed 135 patients who received ECT from a single state psychiatric facility. While this report showed a reduced risk of all-cause mortality, baseline differences between the group receiving ECT and the group that did not receive ECT were not accounted for.[9] Another study from the state of New York reported a higher mortality risk among ECT patients; again, there was no attempt to account for baseline clinical differences between cohorts.[10]

Clinical practice standards suggest ECT should be considered for patients with mood disorders at risk of suicide.[11–13] However, the evidence of the effects of ECT on suicide deaths have been inconsistent.[14] While some studies suggest a protective effect of ECT against suicide,[15,16] other studies report that any protective effect is quite transient.[17] Given the nature of ECT, blinded studies are no longer possible. In addition, as ECT is most commonly given to the most severely ill patients, observational studies that sufficiently account for potential confounding factors are crucial to further elucidating this relationship.

In the last few years, a number of high-quality longitudinal studies examining the relationship between ECT, all-cause mortality and suicide using large databases have been published.[17–20] In the present study, we conduct a systematic review and meta-analysis that include these recent large and high-quality studies to better clarify the effects of ECT on all-cause mortality and suicide deaths among patients with psychiatric conditions.

### Methods

### Search strategy

The protocol pertaining to this study was registered on PROSPERO (CRD42025641749). A systematic search was conducted from inception to January 21, 2025. The following databases were systematically searched: PubMed/MEDLINE, PsycINFO, the Cochrane Library, and Embase using Medical Subject Headings (MeSH) terms and text keywords. We also manually searched all relevant studies in GoogleScholar (https://scholar.google.com/). No language restrictions were imposed. Search strategies are provided in **Supplementary Table 1** in the Supplement. Two authors (TGR and SRS) independently identified relevant studies among the results of the systematic search. References of included studies were also screened for relevant studies. This study followed the preferred reporting items for meta-analysis of observational studies in epidemiology (MOOSE) reporting guidelines (**Supplementary Table 2**).[21] Our study used publicly available data and did not include human participant research. As per 45 CFR § 46.102(f), this study was not submitted for institutional review board approval and did not require informed consent procedures.

## Eligibility criteria and study selection

Inclusion criteria were established prior to article reviews and were as follows: (1) patients with a diagnosis of mental disorder using standardized diagnostic criteria (e.g., *Diagnostic and Statistical Manual of Mental Disorders, fifth Edition* [DSM-5] or *International Statistical Classification of Diseases and Related Health Problems, Tenth Revision* [ICD-10]); (2) intervention/comparator groups consisted of ECT compared with placebo, usual care, or another intervention; (3) outcome measures of validated all-cause mortality or suicide deaths; and (4) randomized controlled trial or longitudinal cohort study designs where the aforementioned interventions preceded the observations of outcome measures. Exclusion criteria were as follow: (1) non-human studies; (2) no use of standardized measures for mental disorder or primary outcomes of interest; or (3) case-control or anecdotal (e.g., case series, case reports, and editorials) study designs. Within-group comparisons (i.e., when participants were receiving the ECT compared to when they were not) were excluded, as were studies using historical comparisons.

### Study identification and data extraction

Titles and abstracts were independently screened by two reviewers (TGR and SRS), and articles identified as potentially relevant by at least one reviewer were retrieved and duplicates were removed. Full-text articles were independently screened by the same reviewers, and discrepancies were resolved through discussion. Data from included articles were independently extracted by the two reviewers using a pilot-tested data extraction form and then corroborated, with discrepancies resolved through discussion. Information to be extracted was established a priori and included: study characteristics (e.g., PICOTS framework), participant characteristics (e.g., clinical and diagnostic information as well as other co-morbidities), data source, selection of cases and controls, sample size and follow-up period, modes of ascertainment, methods of data analysis (e.g., relative measures using adjusted odds ratio [OR], hazard ratio [HR], or relative risk [RR]), and quantitative data pertaining to any primary and secondary outcomes along with covariates. For observational studies where cohort groups often differed with respect to person-time of exposure, we favored (where available) the use of person-years in the denominator. To ensure the absence of overlapping data and to maintain the integrity of the meta-analysis, data and references for each included study were carefully cross-checked.

### Assessment methodological quality

The risk of bias and methodological quality were evaluated using the Newcastle-Ottawa Scale (NOS) for the assessment of the quality of non-randomized studies in meta-analyses.[22,23] We assessed eight items across three domains: selection (adequate definition of case; representativeness of case; definition of control; selection of control); comparability of cases and controls based on the design or analysis; and exposure/outcome (ascertainment of exposure or outcome; same method of ascertainment for cases and controls; and non-response rate). The overall quality was classified as being indicative of a '*good quality*,' '*fair quality*,' or '*poor quality*.'[22,23]

### Publication bias

We used funnel plots to visually explore for evidence of association between the effect sizes and statistical precision.[24] We supplemented visual assessments with statistical testing of funnel plot asymmetry using Egger's test (i.e., a weighted linear regression of effect size versus precision)[25] and Begg and Mazumdar's test (i.e., rank correlation test).[26] Evidence of associations between effect sizes and precision across studies may indicate design heterogeneity, chance or selection biases that operate cross the evidence base (e.g., publication and outcome reporting bias).

## Statistical analysis

The primary outcomes of interest for each comparison were (1) death by any cause (i.e., all-cause mortality) and (2) suicide deaths, assessed as described in each study (either through prospective observation or database analysis [e.g., the Nation Death Index or similar registries]).

Where possible, the time over which the outcomes were assessed was standardized across studies (e.g., 3-, 6-, and 12-month follow-up periods). Because different studies reported three types of relative measures (i.e., OR, HR, and RR), we converted adjusted ORs and RRs to HRs. The weight of each study was determined using an inverse-variance method.[24]

We used both *I*-squared statistic and Cochran's *Q*-statistic to quantify the proportions of heterogeneity due to within- and between-study variations.[24] To adequately estimate the overall effect sizes, relative measures (e.g., ORs, HRs, or RRs) with their corresponding 95% confidence intervals (CIs) were calculated using fixed- or random-effects models depending on the model assumptions.[24] More specifically, a random-effects model was used when *I*-squared statistic was > 50% and a fixed-effects model was used when *I*-squared statistic was < 50%. We reported both random- and fixed-effects models when *I*-squares statistics was 50%.

The *Q*-statistic was used to assess between-study heterogeneity. This statistic provides a quantitative test indicating whether different effect sizes between studies are attributable to subject-level sampling alone or other sources of variability. We estimated the degree of heterogeneity using the *I*-squared statistic, which estimates the proportion of total variance attributable to variance between different studies.

We also conducted moderator analyses using meta-regression analyses by study sample size; relative risk measure (e.g., OR, HR, and RR); age; female sex (%); diagnosis (including psychotic depression and bipolar depression); and country. When identifying potential moderators, we used the variance of the true effects using a restricted maximum likelihood (REML) estimator.[27] We used a statistical software, R 4.2.1. [R Foundation for Statistical Computing] for all analyses using the "*meta*" package.[28] Finally, as mentioned earlier, we assessed publication bias (or small-study effects) using funnel plots;[24] Egger's test and Begg and Mazumdar's test were also performed when assessing the publication bias.[24] Unless otherwise noted, a two-sided *p*-value < 0.05 was considered for statistically significance.

### Role of the funding source

The funders were not involved in the study design, data collection, analysis, interpretation of data, writing of the report, or the decision to submit the manuscript for publication.

#### RESULTS

### Characteristics of included studies

The literature search yielded 7,530 articles, of which 63 were eligible after screening titles and abstracts and removing duplicates. Of these eligible studies, 54 were further excluded after full-text screening. Two independent investigators (TGR and SRS) discovered 8 additional studies by manually searching reference lists and GoogleScholar (Fig. 1). Overall, 17 studies[9,10,15–20,29–39] consisting of 1,182,501 subjects (n = 40,867 for patients receiving ECT and n = 1,141,634 for patients receiving other comparable interventions) were included in the review (Table 1). From these studies, 13 effect sizes for all-cause mortality and 12 effect sizes for suicide deaths were identified for meta-analyses. Fourteen of 17 studies (82.4%) were conducted in inpatient settings, and 9 of 17 (52.9%) were conducted in the United States. Almost all studies had patients with unipolar or bipolar depression, except one study[31] that had patients with schizoaffective disorder. Table 1 provides details of study characteristics, including mean age, proportion of female sex and follow-up time, for all included studies.

Table 1
Selected characteristics of included studies for both qualitative and quantitative analyses

| Author and publication year | Diagnosis | Sample size (ECT) | Sample size (control) | Inpatient v. outpatient | Country | Mean age | Proportion female | Proportion psychotic depression | Follow Up Time |
|---|---|---|---|---|---|---|---|---|---|
| Huston et al., 1948[29,30] | MDD/bipolar depression | 74 | 80 | Inpatient | USA | Varies | 56.5% | 29.9% | 35 months (median) |
| Avery et al., 1978[9] | Depression | 257 | 334 | Inpatient | USA | Unknown | 62% | 20.2% | Six months |
| Tsuang et al., 1979[31] | Schizoaffective disorder | 24 | 50 | Inpatient | USA | 29.6 (ECT) and 30.5 (C) | 71.4% | Unknown | 27 years (mean) |
| Babigian et al., 1984[10] | Mixed depression | Varies | Varies | Mixed | USA | Varies | Varies | Varies | 14 years |
| Philibert et al, 1995[32] | Unipolar depression | 108 | 84 | Inpatient | USA | 72.1 (ECT) and 70.9 (C) | 64.8% | 32.4% | 11 years |
| Munk-Olsen et al., 2007[33] | Mixed | 20,923 person-years | Unknown | Inpatient | Denmark | Unknown | Unknown | Unknown | 25 years |
| *Nordenskjold et al., 2013[34] | MDD | 28 | 28 | Inpatient | Sweden | 52 (ECT) and 62 (C) | 57% | 36% | 1 year |
| Ahmadi et al,, 2016[16] | MDD/ PTSD | 92 | 3,393 | Inpatient | USA | 52 (ECT) and 53 (C) | 14.0% | Unknown | 8 years |
| Liang et al., 2017[35] | Mixed | 1,571 | 827,328 | Inpatient | USA | 42.1 (ECT) and 54.4 (C) | 57.5% | Unknown | Up to 16 years |
| Liang et al., 2018[36] | MDD/bipolar depression | 487 | 1,948 | Inpatient | Taiwan | 38.5 (ECT) and 39.2 (C) | 64.3% | Unknown | 4.4 years (median) |
| Jorgensen et al., 2020[37] | MDD | 5,004 | 87,891 | Mixed (38.0% inpatient) | Denmark | Ranged from 10 to 101 | 62.5% | 5.1% | Variable |
| Osler et al., 2021[19] | MDD/bipolar depression | 6,943 | 167,552 | Mixed | Denmark | Ranged from < 40 to > 80 | 62.0% | 3.1% | 6.7 years |
| Rhee et al., 2021[17] | MDD | 10,460 | 31,160 | Inpatient | USA (Medicare) | 74.7 on average | 65.3% | 1.0% | 1 year |
| Ronnqvist et al., 2021[18] | MDD | 5,525 | 5,525 | Inpatient | Sweden | 55.9 (ECT) and 45.2 (C) | 58.0% | 23.8% | 1 year |
| Watts et al., 2021;[38]2022[47] | Mixed | 5,097 | 10,097 | Inpatient | USA (VA) | 55.7 (ECT) and 55.9 (C) | 12.3% | Unknown | 1 year |
| Kaster et al., 2022[20] | MDD/bipolar depression | 4,982 | 5,304 | Inpatient | Canada | 57.5 (ECT) and 57.1 (C) | 65.9% | 21.1% | 1 year |
| Yamazaki et al., 2022[39] | Bipolar depression | 215 | 860 | Inpatient | Japan | 55.4 (ECT) | 61.0% | 28.8% | 1 year |

| Author and publication year | Diagnosis | Sample size (ECT) | Sample size (control) | Inpatient v. outpatient | Country | Mean age | Proportion female | Proportion psychotic depression | Follow Up Time |
|---|---|---|---|---|---|---|---|---|---|
| | | | | | | and 56.3 (C) | | | |

## All-cause Mortality

Overall, 13 studies[16–20,29–35,38,39] reported the effects of ECT on all-cause mortality (Fig. 2). When these 13 effect sizes were meta-synthesized, patients who received ECT had a lower risk of all-cause mortality (HR, 0.70 [95% CI, 0.61–0.81]; k = 13; $p < 0.001$). The cumulative forest plot by publication year is reported in **Supplementary Fig. 1**. When stratified by specified follow-up times, this association was significant at 3 months (HR, 0.53 [95% CI, 0.39–0.72]; k = 4; $p < 0.001$), 6 months (HR, 0.64 [95% CI, 0.55–0.73]; k = 5; $p < 0.001$), and 12 months (HR, 0.72 [95% CI, 0.61–0.86]; k = 7; $p < 0.001$) (**Supplementary Figs. 3B, 3C, and 3D**). The association at 1 month of follow-up was of similar magnitude as the association at month 3, but was not statistically significant (HR, 0.49 [95% CI, 0.20–1.18]; k = 3; $p = 0.11$), likely due to fewer studies reporting at this time point.

## Suicide Mortality

Overall, 12 studies[15–18,20,29–31,33,34,36–38] reported the effects of ECT on suicide deaths. When these 12 effect sizes were meta-synthesized, there was no association between ECT and the risk of suicide deaths (HR, 0.83 [95% CI, 0.51–1.34]; k = 12; $p = 0.44$) (Fig. 3). The cumulative forest plot by publication year is reported in **Supplementary Fig. 2**. When stratified by specified follow-up times (**Supplementary Figs. 4A, 4C, and 4D**), an association was observed between ECT receipt and reduced risk of suicide at 3 months (HR = 0.53 [95% CI 0.39, 0.72]; k = 4; $p < 0.001$) (**Supplementary Fig. 4B**) but not at 1 month (k = 2), 6 months (k = 3), or 12 months (k = 8).

## Moderator analysis

We explored potential moderating roles of the following variables using meta-regression (for continuous measures) or meta-ANOVA (for binary or categorical measures) models: study sample size; type of relative risk (i.e., HR, OR, or RR); age; female sex (%); follow-up duration; diagnosis; and country of study. Female sex had a statistically significant but very small moderating effect, with females experiencing a slightly smaller ECT effect on mortality. For all-cause mortality as an outcome of interest, we identified country of study as a potential moderator of outcomes ($p = 0.002$) (Table 2). Each country has reported a lower risk of all-cause mortality, but the effect sizes vary by country. Japan had the largest effect size (HR = 0.17, 95% CI 0.04 to 0.72; k = 1) and Denmark had the smallest (HR = 0.87, 95% CI 0.83 to 0.92, k = 2). We have also reported the findings of moderator analysis for all-cause mortality by the follow-up period in **Supplementary Table 3**. The number of patients enrolled in the component studies was considered a potential moderator of suicide deaths ($p < 0.001$) (Table 3). We have also reported the findings of moderator analysis for suicide deaths by the follow-up period in **Supplementary Table 4**.

Table 2
Moderation analyses of all-cause mortality in ECT

| Moderator | *k* | Coefficient (95% CI) | Hazard ratio (95% CI) | *P*-value |
|---|---|---|---|---|
| Number of total patients | 13 | 0.00 (-0.00 to 0.00) | - | 0.915* |
| Outcome measure | | | | |
| Hazard ratio | 10 | - | 0.65 (0.54–0.79) | 0.081† |
| Odds ratio | 1 | - | 0.87 (0.73–1.03) | |
| Relative risk | 2 | - | 0.72 (0.46–1.12) | |
| Age | | | | |
| Overall | 12 | 0.01 (-0.01 to 0.03) | - | 0.269* |
| 65 or older only | 4 | 0.01 (-0.01 to 0.03) | - | 0.238* |
| Female sex | 11 | -0.01 (-0.01 to -0.00) | - | 0.036* |
| Follow-up duration | 10 | 0.05 (-0.01 to 0.11) | - | 0.125* |
| Condition | | | | |
| % major depressive disorder | 9 | 0.00 (-0.02 to 0.02) | - | 0.985* |
| % psychotic depression | 5 | 0.01 (-0.00 to 0.01) | - | 0.223* |
| % bipolar disorder | 6 | -0.00 (-0.02 to 0.01) | - | 0.506* |
| % psychosis or schizophrenic spectrum disorder | 6 | -0.01 (-0.02 to 0.00) | - | 0.155* |
| Region | | | | |
| United States | 6 | - | 0.52 (0.32–0.83) | 0.002† |
| Canada | 1 | - | 0.75 (0.58–0.97) | |
| Sweden | 2 | - | 0.67 (0.55–0.82) | |
| Denmark | 2 | - | 0.87 (0.83–0.92) | |
| Japan | 1 | - | 0.17 (0.04–0.72) | |
| Taiwan | 1 | - | 0.66 (0.46–0.94) | |

Table 3
Moderation analyses of suicide in ECT

| Moderator | *k* | Coefficient (95% CI) | Hazard ratio (95% CI) | *P*-value |
|---|---|---|---|---|
| Number of total patients | 12 | 0.00 (0.00–0.00) | - | < 0.001* |
| Outcome measure | | | | |
| Hazard ratio | 9 | - | 0.77 (0.40–1.50) | 0.225† |
| Odds ratio | 1 | - | 1.38 (0.95–2.01) | |
| Relative risk | 2 | - | 0.70 (0.21–2.25) | |
| Age | | | | |
| Overall | 10 | 0.01 (-0.05 to 0.06) | - | 0.858* |
| 65 or older only | 3 | 0.01 (-0.01 to 0.03) | - | 0.340* |
| Female sex | 9 | -0.00 (-0.02 to -0.01) | - | 0.639* |
| Follow-up duration | 10 | 0.10 (-0.10 to 0.30) | - | 0.312* |
| Condition | | | | |
| % major depressive disorder | 8 | -0.02 (-0.04 to 0.01) | - | 0.233* |
| % psychotic depression | 4 | -0.01 (-0.03 to 0.01) | - | 0.170* |
| % bipolar disorder | 6 | 0.01 (-0.00 to 0.03) | - | 0.149* |
| % psychosis or schizophrenic spectrum disorder | 6 | -0.00 (-0.21 to 0.21) | - | 0.997* |
| Region | | | | |
| United States | 6 | - | 0.58 (0.29–1.18) | 0.251† |
| Canada | 1 | - | 0.53 (0.31–0.91) | |
| Sweden | 2 | - | 0.72 (0.52-1.00) | |
| Denmark | 2 | - | 2.27 (0.63–8.26) | |
| Taiwan | 1 | - | 0.80 (0.65–0.99) | |

## Methodological quality and publication bias

Methodological quality of the included studies and our justification were provided in **Supplementary Table 5** in the Supplement. Studies published after 2005 were considered '*good*,' and studies published in the 1980's or earlier were considered '*poor*' to '*fair*.' Only one study[34] was a randomized controlled trial, where the risk of bias was considered '*low*.' In our main analysis, we did not find any potential publication bias for all-cause mortality as the primary outcome of interest (**Supplementary Fig. 5**), using Egger's test ($p = 0.10$) and Begg and Mazumdar's test ($p = 0.14$). For suicide deaths as the outcome of interest (**Supplementary Fig. 6**), we did not find any potential publication bias using Egger's test ($p = 0.50$) and Begg and Mazumdar's test ($p = 0.89$).

### DISCUSSION

In this meta-analysis comprising over 41,000 patients that received ECT, it was determined that ECT was associated with a reduction in risk of all-cause mortality, a finding that was consistent at 3 months, 6 months, and 12 months of follow-up. Sex demonstrated a statistically significant but very small moderating effect on this relationship, with ECT being slightly less protective against all-cause mortality risk for females compared to males. Country also had a moderating effect. Each country has reported a lower risk of all-cause mortality associated with ECT, but the effect sizes vary by country. Japan had the largest effect size whereas Denmark had the smallest. ECT was associated with a reduction in suicide risk at 3 months of follow-up but not at 1, 6, or 12 months of follow-up.

The findings of this meta-analysis suggest a treatment effect whereby ECT reduces the risk of all-cause mortality. Possible mechanisms whereby ECT may have a causal protective effect on mortality include both psychological/functional and biological mechanisms. With respect to a psychological/functional mechanism, one potential way in which ECT might lead to reduction in all-cause mortality risk is that ECT may help improve functioning, allowing patients to better engage in treatment for whatever medical conditions they face or maintain a healthier lifestyle. This is supported by a prior report wherein ECT recipients were less likely to die from a variety of causes, including smoking-related causes, cancer, circulatory diseases, and diabetes.[17] With respect to potential biological mechanisms, ECT has been shown to reduce markers of neurodegeneration, and may also help improve innate immune functioning in individuals with depression.[40] Successful treatment of mental illness may also normalize pathological vascular states that have been linked to mood disorders.[41]

Given that most studies included in this meta-analysis were observational in nature, the possibility that confounding explains this association must be considered.[42] It is possible that patients with severe medical illness are not selected for ECT because of the risk of the procedure. We judge that confounding cannot account for the whole of the association between ECT and reduced mortality risk. One such reason is because many of the more recent, methodologically sophisticated longitudinal studies show a substantial difference between ECT and non-ECT groups in terms of mortality risk after carefully balancing known medical comorbidities. For instance, Kaster et al., conducted an assessment in which each of a range of 25 serious medical conditions were matched between the groups with a standardized mean difference of 0.02 or less.[20] Rhee et al., conducted a sub-analysis wherein patients who received a subtherapeutic course of ECT (defined to be 4 treatments or fewer) were compared to those receiving a therapeutic course and those who did not receive ECT.[17] The trajectory of mortality risk of those receiving a subtherapeutic course of ECT matched those who did not receive ECT for the first 6 months following hospital discharge (both of these groups were at greater risk of mortality compared to the therapeutic ECT group).[17] Hence, while confounding may explain some of the observed association between ECT utilization and lower risk of all-cause mortality, several lines of evidence suggest that confounding does not explain all of the association.

Previous studies on the possible effects of ECT on suicide have been inconsistent.[43] Notably, treatment guidelines from the American Psychiatric Association as well as the Canadian Network for Mood and Anxiety Treatments recommend the use of ECT for those with acute suicidal ideation.[44] The current meta-analysis did not find a consistent relationship between ECT and suicide risk. Notably, for those studies (k = 4) that specifically reported outcomes 3 months after index date, a significant association was found between ECT receipt and reduced risk of suicide death. No association was found at months 1 (k = 2), 6 (k = 3), or 12 (k = 8). Possibile explanations include discrepancies in power (for 1- and 6-month outcomes) and/or that any protective effect of ECT against suicide wanes beyond 3 months, especially if patients do not continue to receive continuation/maintenance therapy. It has long been shown that relapse rates are quite high (84%) among ECT responders by 6 months if no continuation/maintenance therapy is initiated.[45]

Our analysis suggested that the association between ECT and all-cause mortality is slightly weaker for females than for males (standardized beta coefficient, -0.01). The significance of this finding is uncertain and, to our knowledge, has not been reported previously. This analysis also showed that country also had a moderating effect. Each country has reported a lower risk of all-cause mortality associated with ECT, but the effect sizes vary by country. The significance of this is unknown. Of note, all countries except the United States only had two or fewer studies.

Several limitations of the current study require comment. As noted, confounding cannot be entirely ruled out, as most studies included in this meta-analysis were non-randomized. Additionally, as is usually the case in meta-analyses, studies varied in the way outcomes were reported. This resulted in small numbers of studies for time-specific outcomes (k = 2, for ECT and suicide association at 1 month). Furthermore, most studies did not report on what types of treatment were received at the conclusion of the index course of ECT. It has long been noted that the type of continuation/maintenance therapy can have a large bearing on clinical outcomes.[45,46]

## Conclusions

ECT is strongly associated with a reduced risk of all-cause mortality. This finding is seen internationally, across age groups and sexes. While confounding cannot be entirely ruled out due to most studies being non-randomized, recent large and high-quality studies that controlled for many measures of medical comorbidity affirmed this finding. ECT is not consistently associated with a reduced risk of suicide.

## Declarations

Acknowledgement and disclosures

*Study approval statement*:An ethics statement is not applicable because this study is based exclusively on published literature.

*Consent to participate statement*: Written informed consent was not required as this study is based exclusively on published literature.

*Data availability statement*: Data are publicly available. All data generated or analyzed during this study are included in this article. Further enquiries can be directed to the corresponding author.

*Author contributions*: Study concept and design: TGR; Data acquisition and statistical analyses: TGR & SRS; Interpretation of data: TGR, SRS, MN, RSM, TSK, & STW; Drafting of manuscript: TGR, SRS, MN, & STW; Critical revision of manuscript for important intellectual content: TGR, SRS, MN, RSM, TSK, & STW; Supervision: TGR & STW. TGR and SRS are co-first authors. TGR had full access to all the data in the study and takes responsibility for the integrity of the data and the accuracy of the data analysis.

*Role of the funder/sponsor*: This study was not funded.

*Conflict of interest disclosures*: Each author completed and submitted the ICMJE Form for Disclosure of Potential Conflicts of Interest and had none directly related to this manuscript.

1. **Rhee** was supported in part by the National Institute on Aging (NIA) (#R21AG070666; R21AG078972), National Institute of Mental Health (#R21MH117438), National Institute on Drug Abuse (#R21DA057540) and Institute for Collaboration on Health, Intervention, and Policy (InCHIP) of the University of Connecticut. Dr. Rhee serves as a review committee member for Patient-Centered Outcomes Research Institute (PCORI) and Substance Abuse and Mental Health Services Administration (SAMHSA) and has received honoraria payments from PCORI and SAMHSA. Dr. Rhee has also served as a stakeholder/consultant for PCORI and received consulting fees from PCORI. Dr. Rhee serves as an advisory committee member for International Alliance of Mental Health Research Funders (IAMHRF).

2. **Shim** reported none.

3. **Nasir** reported none.

4. **McIntyre** has received research grant support from CIHR/GACD/National Natural Science Foundation of China (NSFC); speaker/consultation fees from Lundbeck, Janssen, Alkermes, Mitsubishi, Tanabe, Purdue, Pfizer, Otsuka, Takeda, Neurocrine, Sunovion, Bausch Health, Axsome, Novo Nordisk,Kris, Sanofi, Eisai, Intra-Cellular, NewBridge Pharmaceuticals, Abbvie, Atai Life Sciences.

5. **Kaster** is supported by the Canadian Institute for Health Research, the AFP Innovation Fund, and the Patient-Centered Outcomes Research Institute.

**Wilkinson** reported receiving funding from Sage Therapeutics and Oui Therapeutics for the conduct of clinical trials (administered through Yale University) and personal fees from LivaNova and Mind Medicine outside the submitted work.

**Funding:** This study was not funded.

## Figures

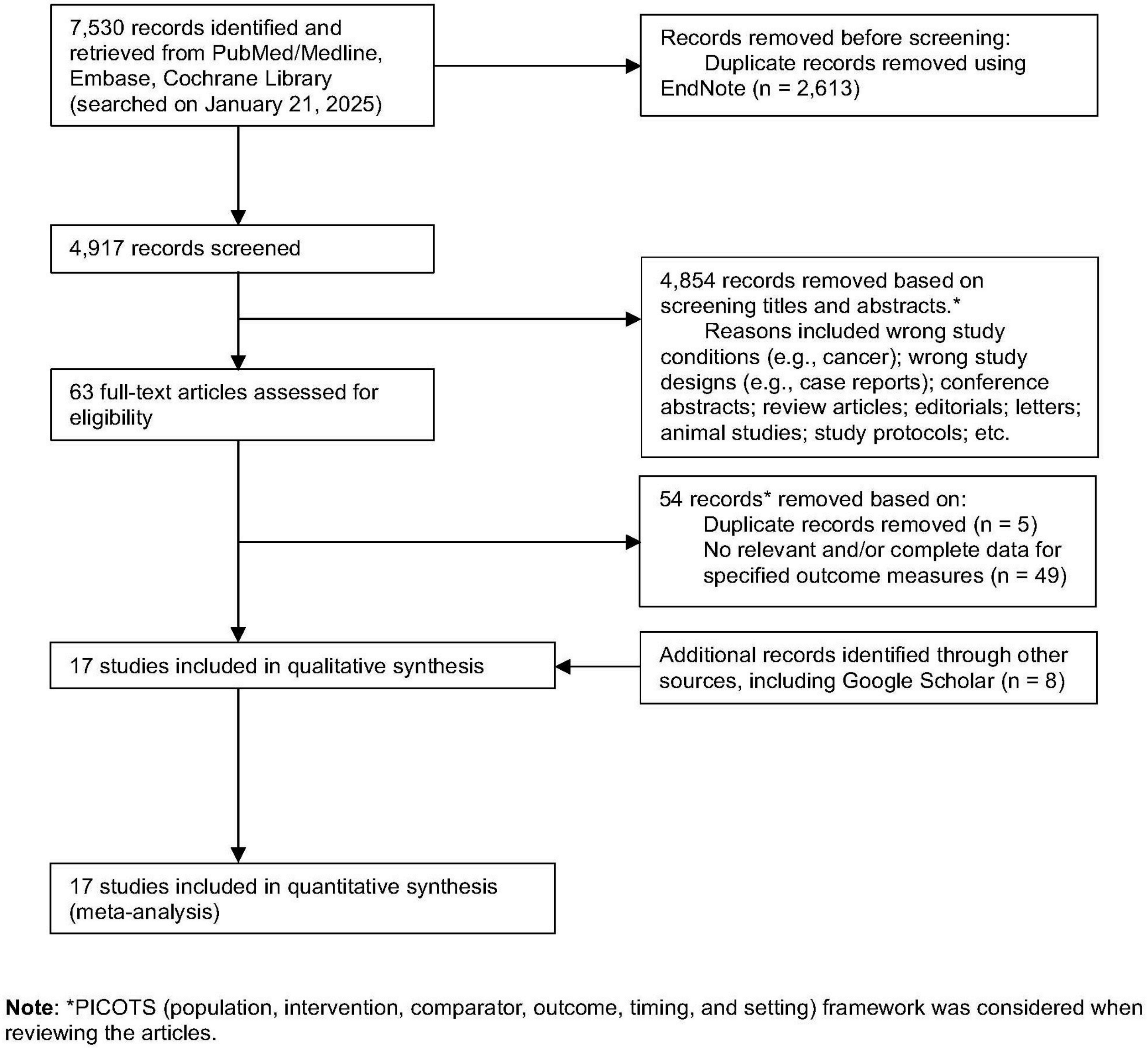

**Figure 1**

Study selection flowchart

**Note**: *PICOTS indicates population, intervention, comparator, outcome, timing, and setting.

| Study | n for ECT | n for Control | Duration | HR | 95% CI | Hazard Ratio IV, Random, 95% CI |
|---|---|---|---|---|---|---|
| Huston (1948) | 74 | 80 | 12 | 0.108 | [0.033; 0.353] | |
| Tsuang (1979) | 24 | 50 | . | 0.245 | [0.094; 0.640] | |
| Philibert (1995) | 108 | 84 | . | 0.798 | [0.511; 1.244] | |
| Munk−Olsen (2007) | 783 | 5781 | . | 0.860 | [0.790; 0.940] | |
| Nordenskjold (2013) | 28 | 28 | 12 | 1.000 | [0.020; 50.400] | |
| Ahmadi (2016) | 92 | 3393 | 3 | 0.540 | [0.340; 0.860] | |
| Liang (2017) | 1571 | 827328 | 12 | 0.656 | [0.460; 0.936] | |
| Rhee (2021) | 10460 | 31160 | 12 | 0.610 | [0.560; 0.660] | |
| Ronnqvist (2021) | 5525 | 5525 | 12 | 0.670 | [0.550; 0.820] | |
| Osler (2021) | 6943 | 167552 | 12 | 0.879 | [0.830; 0.931] | |
| Watts (2021) | 5097 | 10097 | 12 | 0.870 | [0.790; 1.110] | |
| Kaster (2022) | 4982 | 5304 | 12 | 0.750 | [0.580; 0.970] | |
| Yamazaki (2022) | 215 | 860 | 3 | 0.174 | [0.042; 0.716] | |
| **Total (95% CI)** | | | | **0.703** | **[0.612; 0.808]** | |

Heterogeneity: $Tau^2 = 0.0333$; $Chi^2 = 83.75$, df = 12 ($P < 0.01$); $I^2 = 86\%$

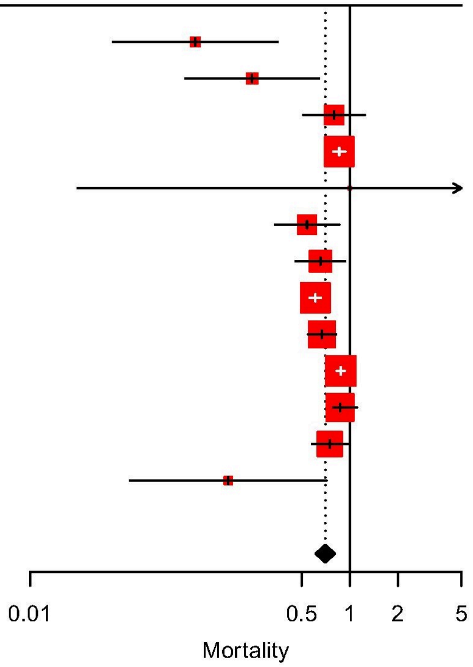

**Figure 2**

Forest plot of all-cause mortality in ECT

**Note**: A hazard ratio of <1.0 indicates a reduction of suicide deaths in the ECT group (i.e., favors ECT).

| Study | n for ECT | n for Control | Duration | HR | 95% CI |
|---|---|---|---|---|---|
| Huston (1948) | 74 | 80 | 12 | 0.180 | [0.041; 0.794] |
| Avery (1978) | 257 | 192 | 12 | 0.249 | [0.015; 4.101] |
| Tsuang (1979) | 24 | 50 | . | 0.298 | [0.037; 2.415] |
| Munk−Olsen (2007) | 783 | 5781 | . | 1.200 | [0.990; 1.470] |
| Nordenskjold (2013) | 28 | 28 | 12 | 1.000 | [0.020; 50.400] |
| Ahmadi (2016) | 92 | 3393 | 3 | 0.360 | [0.100; 0.450] |
| Liang (2018) | 487 | 1948 | 12 | 0.803 | [0.621; 0.938] |
| Jorgenson (2020) | 5004 | 87891 | 12 | 4.480 | [2.280; 6.140] |
| Rhee (2021) | 10460 | 31160 | 12 | 0.920 | [0.680; 1.250] |
| Ronnqvist (2021) | 5525 | 5525 | 12 | 0.720 | [0.520; 0.990] |
| Watts (2021) | 5097 | 10097 | 12 | 1.380 | [0.880; 1.870] |
| Kaster (2022) | 4982 | 5304 | 12 | 0.530 | [0.310; 0.920] |
| **Total (95% CI)** | | | | **0.827** | **[0.510; 1.341]** |

Heterogeneity: $Tau^2 = 0.5134$; $Chi^2 = 69.53$, df = 11 ($P < 0.01$); $I^2 = 84\%$

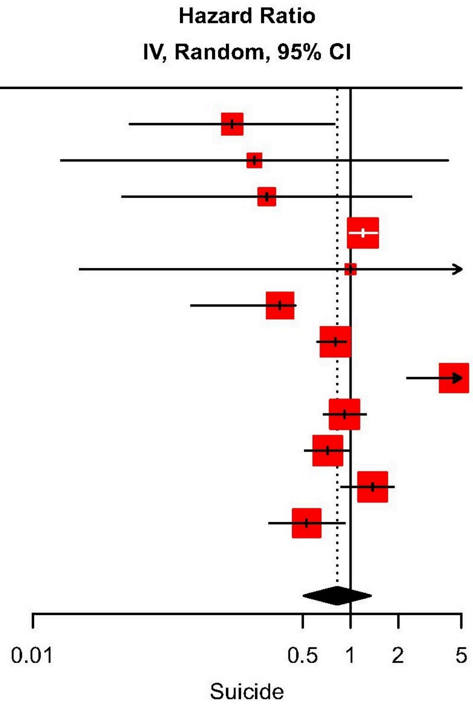

**Figure 3**

Forest plot of suicide mortality in ECT

**Note**: A hazard ratio of <1.0 indicates a reduction of suicide deaths in the ECT group (i.e., favors ECT).

### Supplementary Files

This is a list of supplementary files associated with this preprint. Click to download.

- ECTandmortalityMASupplement372025.docx
